# Characterization of oxidation of glutathione by cytochrome c

**DOI:** 10.1007/s10863-021-09926-z

**Published:** 2021-12-10

**Authors:** K. B. Csomó, B. Alasztics B, A. P. Sándor, A. A. Belik, G. Varga, A. Hrabák, Z. Kukor

**Affiliations:** 1 Department of Biochemistry and Molecular Biology, Institute of Molecular Biology, Semmelweis University, Tűzoltó u. 37-47, Budapest, 1093 Hungary; 2 Department of Conservative Dentistry, Semmelweis University, Szentkirályi u. 47, Budapest, 1088 Hungary; 3 Department of Oral Biology, Semmelweis University, Nagyvárad tér 4, Budapest, 1089 Hungary

**Keywords:** Cytochrome c, Glutathione, Mitochondria

## Abstract

Cytochrome c is a member of the respiratory chain of the mitochondria. Non-membrane-bound (free) cytochrome c can be reduced by gluthatione as well as ascorbic acid. We investigated the effect of pH, Ca^2+^, Mg^2+^ and anionic phospholipids on the reduction of cytochrome c by glutathione.The reduction of cytochrome c by thiols was measured using photometry. Mitochondrial oxygen consumption was detected by use of oxygen electrode. Glutathione does not reduce cytochrome c at pH = 7.0 in the absence of Ca^2+^ and Mg^2+^. The reduction of cytochrome c by glutathione is inhibited by anionic lipids, especially cardiolipin. The typical conditions of apoptosis—elevated pH, Ca^2+^ level and Mg^2+^—increases the reduction of cytochrome c. Glutathione (5 mM) causes increased mitochondrial O_2_ consumption at pH = 8.0, in the presence of ADP either 1 mM Mg^2+^ or 1 mM Ca^2+^. Our results suggest that membrane bound cyt c does not oxidize glutathione. Free (not membrane bound) cytochrome c can oxidize glutathione. In mitochondria, O_2_ is depleted only in the presence of ADP, so the O_2_ depletion observed in the presence of glutathione can be related to the respiratory chain. Decreased glutathione levels play a role in apoptosis. Therefore, membrane unbound cyt c can contribute to apoptosis by oxidation of glutathione.

## Introduction

In healthy cells, the primary function of mitochondria is ATP production by the respiratory chain. Cytochrome c (cyt c) is a member of the respiratory chain proteins, and it is located between complex III and complex IV in the intermembrane space of the mitochondrion. In vivo, electrons go to complex III from NADH or from succinate and fatty acids.

The primary function of cyt c is electron shuttling in the oxidative phosphorylation process.

In physiological conditions cyt c is bound to the inner mitochondrial membrane. At physiologic pH, cyt c is a positively charged protein; thus it mainly binds to anionic lipids such as phosphatidyl-serine or mitochondric-specific cardiolipin through electrostatic and hydrophobic interactions (Ott et al. 2002). Cyt c has an important role in the terminal oxidation process, as well as in the electron transport. The unbound part of cyt c diffuses primarily in the intermembrane space of intact mitochondria at physiological ionic strength (Cortese et al. 1995). Thus in agreement with its role as a soluble, three-dimensional diffusant in the intermembrane space of mitochondria, the cyt c carries electrons from the membrane-bound Complex III to cyt c oxidase (Cortese et al. 1995; Gupte and Hackenbrock 1988).

However, under pro-apoptotic conditions, including oxidative stress, cyt c translocates into the cytosol to engage in the intrinsic apoptotic pathway, and enters the nucleus where it impedes nucleosome assembly. Other reported functions include cytosolic redox sensing and involvement in the mitochondrial oxidative folding machinery (Alvarez-Paggi et al. 2017).

Cyt c can be released from the intermembrane space. The release of cyt c from the mitochondria is a key step in the mitochondrion-derived apoptosis. The first step of the release of cyt c is termination of the bond between cyt c and cardiolipin. Oxidation of cardiolipin is necessary in order to rupture the bond with the tightly bound cytochrome c. Cyt c leaves the intramitochondrial space through the permeability transition pore (PTP), but the mechanism of this step is not entirely clear.

In vitro, cyt c is reduced by miscellaneous reduction agents such as dithiothreitol, glutathione (GSH) or ascorbic acid (Williams and Yandell 1985).

GSH is an important antioxidant; the concentration levels in mammalian cells are in the millimolar range (1–10 mM). The synthesis of GSH from its constituent amino acids occurs exclusively in cytosol. GSH can cross the outer mitochondrial membrane. GSH concentration of mitochondrial intermembrane space is similar to that found in the cytosol (depending on the tissue type). Its transport into the mitochondrial matrix cannot be explained by simple diffusion. Therefore, matrix GSH arises from the cytosol GSH by the activity of specific carriers (Ribas et al. 2014). The GSH levels in the mitochondrion is similar to the GSH levels in the cytosol (Pastore et al. 2003), though mitochondrial GSH concentration is higher than cytosolic (Wahlländer et al. 1979). In the intracellular environment GSH is primarily reduced, the rate of GSH:GSSG is 300–30:1, except in the endoplasmic reticulum, where the ratio is 3–1:1. In cardiomyocytes, the intermediate GSH:GSSG ratios may cause reversible mitochondrial ΔΨm, while strongly decreased ratios can cause irreversible PTP activation (Aon et al. 2007).

Cyt c is found in the mitochondria bound to the inner membrane as well as in the intermembrane space. Due to the oxidizing effect of GSH and ascorbic acid, does it arise whether these reactions can take place in the mitochondria? Can the reduction of cyt c (oxidation of glutathione) be related to free (solved) or membrane-bound cyt c? Our main goal was to study the chemical reaction. The intermembrane space was modeled with free cyt c, while membrane-bound cytochrome c was modeled with phospholipids. Using rat liver mitochondria, we investigated whether mitochondrial oxygen consumption could change in the presence of GSH.

## Materials and methods

### Cyt c reduction

The reduction of cytochrome c was determined by absorbance at 550 nm (ɛ = 21,000 1/mol). The typical incubation mixture is composed of 100 µM bovine cytochrome c; 40 mM Tris/HCl (pH 8.0; 7.4 or 7.0) or NaHCO_3_ (pH 6.0 and 6.5); 1 mM GSH; CaCl_2_, MgCl_2_, concentration was as indicated. The micromolar calcium concentration was regulated by the Ca-EDTA complex (Bártfai 1979).

### Preparation of mitochondria

Mitochondria were prepared from 180–200 g Wistar rat liver using a standard protocol from Clayton and Shadel (Clayton and Shadel 2012). Animal experiments were performed in accordance with the Guidelines for Animal Experiments of Semmelweis University. A modified biuret method was used to determine mitochondrial protein concentration (Bradford 1976).

### Mitochondrial oxygen consumption assay

Respiratory rates were determined by measuring the oxygen consumption of mitochondria using Clark type electrode in 1 ml sealed chamber, which was stirred at 37 °C. The mitochondria (1 mg protein/ml) were incubated in 250 mM sucrose, 40 mM Tris/HCl (pH 8.0, pH 7.4 and pH 7.0) or 40 mM NaHCO_3_ (pH 6.2, 6.5); 5 mM glutathione. MgCl_2_, CaCl_2_, EDTA, ADP concentrations were as indicated.

### Data analysis

Testing for statistically significant differences (p < 0.05) was performed by analysis of variance (ANOVA). Data are given as mean ± SD for three-six separate experiments. Authors can confirm that all relevant data are included in the article and data will be made available on reasonable request.

### Materials

Phosphatidyl-serine, phosphatidyl acid, horse Cytochrome c, glutathione, cardiolipin were from Sigma-Aldrich Kft. (Budapest, Hungary). Other chemicals were from Reanal (Budapest, Hungary).

## Results

Free (without added phospholipids) cyt c was model of cyt c in the intermembrane space and cyt c + phospholipids was model of intermembrane bound cyt c. We tested whether glutathione could increase mitochondrial O_2_ intake, so could glutathione act as a respiratory substrate?

### Free (model of intermembrane space) cyt c

#### Cyt c reduction by glutathione depends on pH, Ca^2+^ and Mg^2+^ concentration

**Fig. 1:**
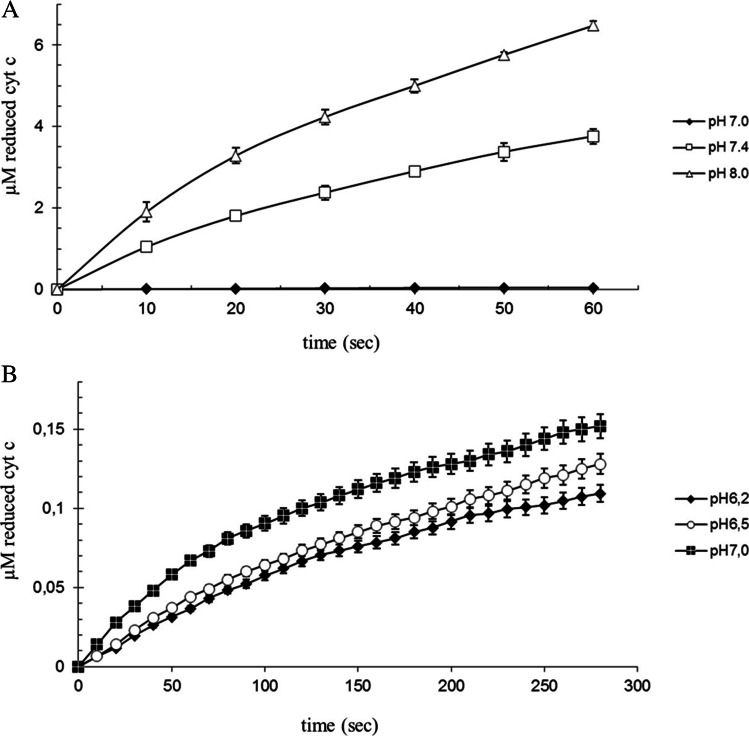
Cyt c reduction by GSH at differently pH. 100 µM bovine cytochrome c, 1 mM GSH, 1 mM EDTA at 25 °C. A pH = 7.0, 7.4 and 8.0 (40 mM Tris/HCl). B pH = 6.0, 6.5 (40 mM NaHCO_3_) and 7.0 (40 mM Tris/HCl). The reaction was started by adding cytochrome c (n = 6 ± SD)

In agreement with previous observations (Hancock et al. 2001), glutathione reduces free cyt c. The reduction rate is dependent on pH and Ca^2+^ concentration. At pH 6.2, 6.5 and 7.0 in 1 mM EDTA cyt c reduction is lightly increases within 300 s at 25 °C. At elevated pH levels, cytochrome c is reduced by GSH. The rate of reduction of cytochrome c by GSH notably grows above pH 7.0 (Fig. 1A and B).

**Fig. 2:**
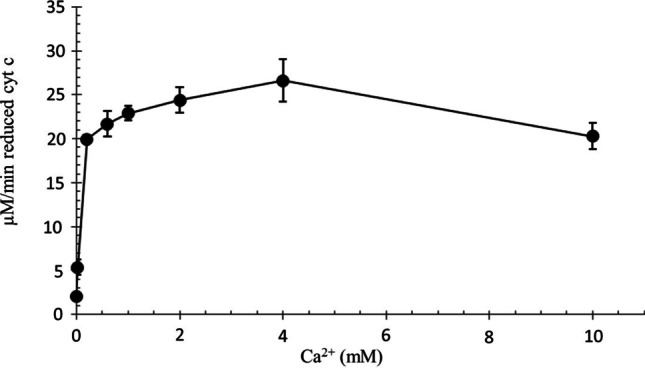
Cyt c reduction by GSH at differently concentrations of Ca^2+^. 100 µM bovine cytochrome c, 1 mM GSH, Ca^2+^ concentrations 0–10 mM, 40 mM Tris/HCl (pH = 8.0), at 25 °C. The reaction was started by adding cytochrome c (n = 6 ± SD)

Ca^2+^ has been known to regulate the respiratory chain attached enzymes (pyruvate dehydrogenase, α-ketoglutarate dehydrogenase, isocitrate dehydrogenase, ATP synthase, adenine nucleotide translocase etc.) to control cell death (Giacomello et al. 2007; Brookes et al. 2004). The increase in Ca^2+^ level in the mitochondria is key to cytochrome c release and apoptosis. Rate of reduction of cytochrome c dependents on Ca^2+^ concentration. The maximal reduction rate is measured at 1–4 mM Ca^2+^, while 10 mM Ca^2+^ decreased (not significant) the reduction rate (Fig. 2).

**Fig. 3:**
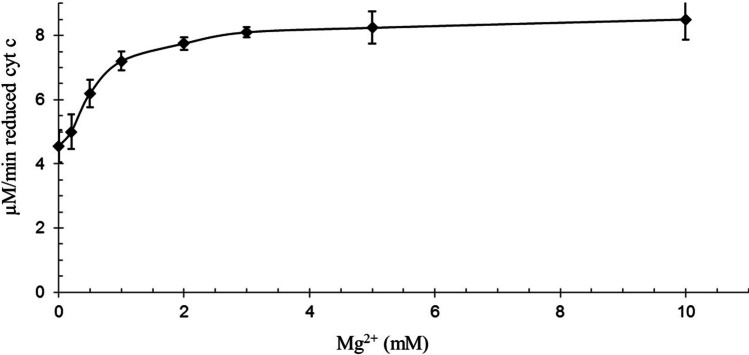
Cyt c reduction by GSH at differently concentrations of Mg^2+^. 100 µM bovine cytochrome c, 1 mM GSH, Mg^2+^ concentrations 0–10 mM 40 mM Tris/HCl (pH = 8.0), at 25 °C. The reaction was started by adding cytochrome c (n = 6 ± SD)

Mg^2+^, as a bivalent cation can modify the reduction of cytochrome c. The reduction of cytochrome c dependents on Mg^2+^ concentration. The maximal reduction rate is measured at 10 mM Mg^2+^ at pH = 8.0 (Fig. 3).

#### The effect of NaCl on reduction of cytochrome c

The effect of the bivalent cations can also be due to the ionic strength, so we examined whether NaCl, in a concentration (1.5 mM) equivalent to the ionic strength of 1 mM CaCl_2_ and MgCl_2_ concentrations, would affect cyt c reduction. The ionic strength of NaCl, in a concentration equivalent to the ionic strength of MgCl_2_ and CaCl_2_, did not affect the reduction of cyt by GSH (Figure does not show it). The effect was independent of pH.

#### The effect of thiols (GSH, Cys, DTT) on the reduction of cytochrome c

**Fig. 4:**
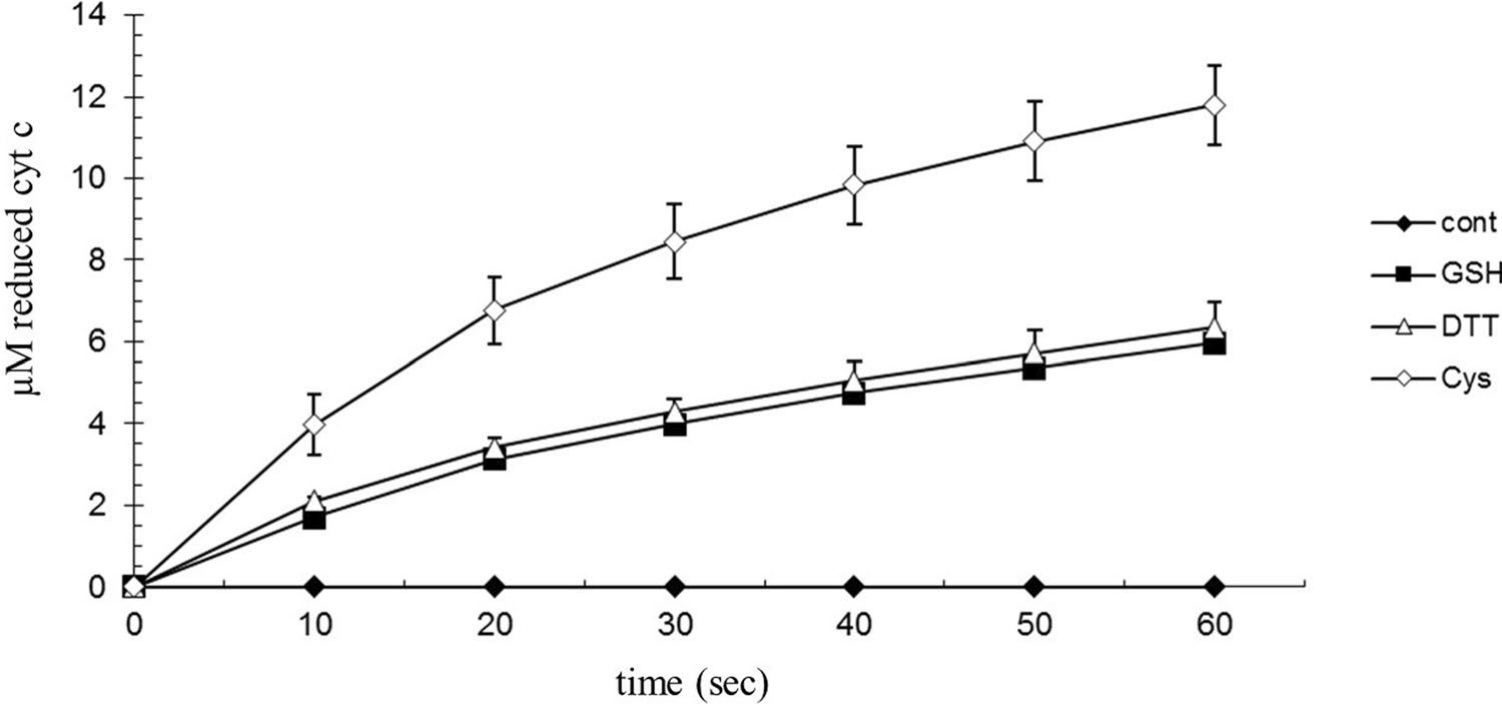
Effect of thiols on the reduction of cytochrome c. 100 µM bovine cytochrome c, 1 mM GSH, 1 mM cysteine (Cys), 0.5 mM (1 mM thiol) dithiothreitol (DTT), 40 mM Tris/HCl (pH = 8.0), at 25 °C. The reaction was started by adding cytochrome c (n = 6 ± SD)

GSH is the thiol compound of largest concentration in the cells. We investigated if cysteine and dithiothreitol (DTT) can reduce cyt c. At the same thiol concentration (1 mM) rate the cyt c reduction was at least double that of cysteine than GSH and DTT (pH 7.0 and pH 8.0; T = 25 °C; p < 0.05) (Fig. 4). The ratio of Cys:GSH the cyt c reduction rate was independent from Ca^2+^.

### Model of intermembrane bound cyt c

#### Negative phospholipids decrease the cytochrome c reduction of glutathione

Under physiological conditions, cytochrome c is not reduced by GSH. The cyt c is attached to CL with ionic and/or hydrogen and hydrophobic bonds.

**Fig. 5:**
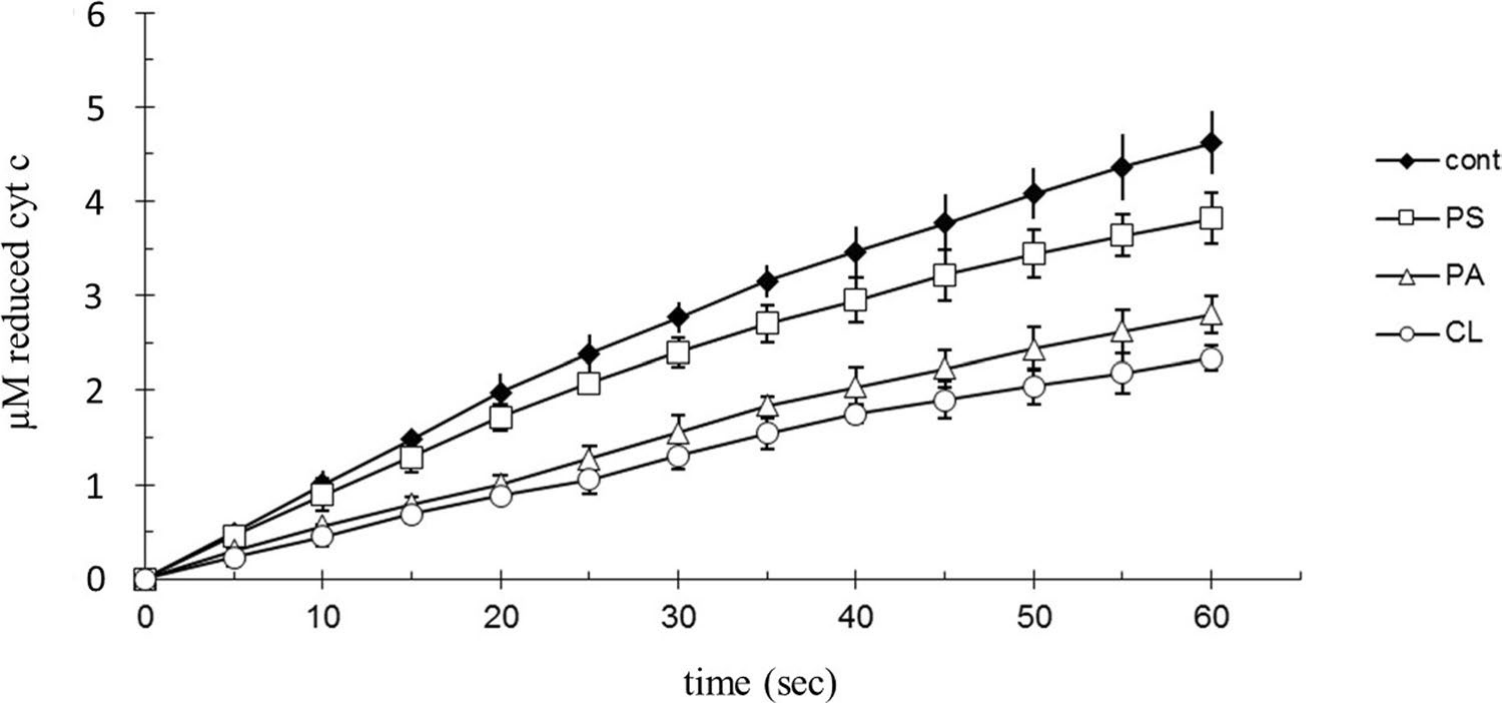
Effect of phospholipids on the reduction rate of cytochrome c by GSH. 100 µM bovine cytochrome c, 40 mM Tris/HCl (pH = 8.0); 0.1 mM EDTA, at 25 °C; 250 µg/ml phosphatidic acid (PA); 250 µg/ml phosphatidyl-serine (PS); 250 μg/ml cardiolipin (CL) 1 mM GSH; 5 v/v% ethanol (phospholipid solvent). The reaction was started by adding cytochrome c

The fastest reduction of free cyt c by GSH was measured at pH = 8.0. Anionic phospholipids (phosphatidic acid (PA), phosphatidyl-serine (PS), cardiolipin (CL)) decrease the rate of cytochrome c reduction. The inhibitor effect is concentration dependent. PS has the poorest inhibitor effect, while CL has the strongest effect (Fig. 5) at a phospholipid concentration of 250 μg/ml.

#### The effect of Ca^2+^, Mg^2+^ and phospholipids on the reduction rate of cytochrome c by GSH

**Fig. 6:**
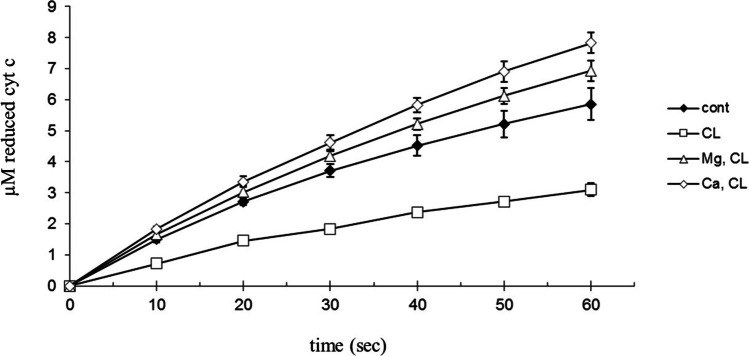
Effect of Ca^2+^, Mg^2+^ and phospholipids on the reduction rate of cytochrome c by GSH. 100 µM bovine cytochrome c; 40 mM Tris/HCl (pH = 8.0); 5 v/v% ethanol (cardiolipin solvent); 1 mM GSH; at 25 °C. 250 μg/ml cardiolipin (CL); 25 μg/ml cardiolipin and 1 mM Ca^2+^ (Ca, CL); control (cont) was without cardiolipin, Ca^2+^, Mg^2+^. The reaction was started by adding cytochrome c

Using a concentration of 250 μg/ml anionic phospholipids to model the membrane bound cyt c, 1 mM Ca^2+^ or Mg^2+^ increases the reduction rate of cytochrome c (Fig. 6). The inhibitor effect of anionic phospholipids on the reduction of cytochrome c can be decreased by bivalent cations (Ca^2+^, Mg^2+^). Isosmotic NaCl (1.5 mM) did not affect the reduction of cyt c by GSH at 250 µg/ml anionic phospholipids concentration.

#### GSH as oxygen consumption substrate of mitochondria

**Fig. 7:**
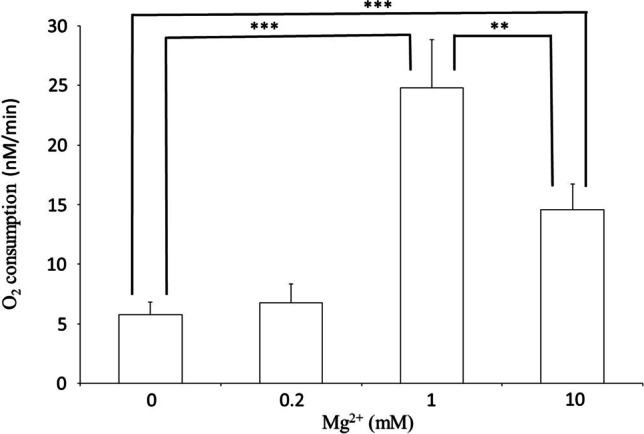
Effect of Mg^2+^ on O_2_ consumption of mitochondria. 80 mM KCl; 20 mM TRIS/HCl; 0,1 mM EGTA; 10 mM KH_2_PO_4_; pH 8.0; 37 °C; 5 mM GSH; 1 mg mitochondrial protein/ml

GSH do not change the basic O_2_ consumption of rat liver mitochondria at pH 7.0 and 1 mM EDTA without added Mg^2+^ or Ca^2+^. The O_2_ consumption of rat liver mitochondria increases at pH 8.0; 5 mM GSH. Mg^2+^ increases the oxygen consumption of the mitochondria in a concentration-dependent manner; the maximal effect was measured at a concentration level of 1 mM Mg^2+^ (Fig. 7). O_2_ consumption is observed only in the presence of ADP (333 μM). In the absence of ADP, mitochondrial O_2_ consumption cannot be increased with Mg^2+^ either.

**Fig. 8:**
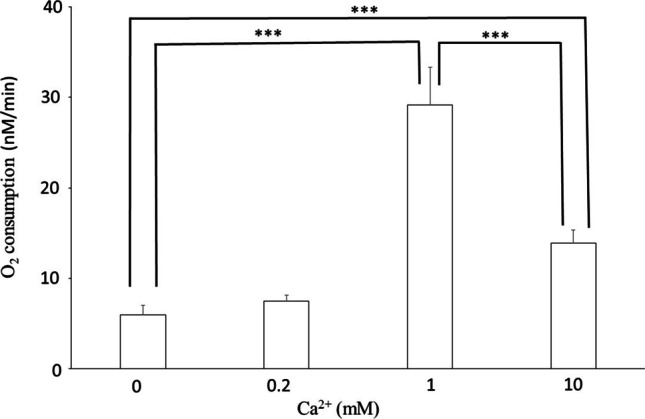
Effect of Ca^2+^ on O_2_ consumption of mitochondria. 80 mM KCl; 20 mM TRIS/HCl; 0,1 mM EGTA; 10 mM KH_2_PO_4_; pH 8.0; 37 °C; 5 mM GSH; 1 mg mitochondrial protein/ml

Ca^2+^ has a similar effect as Mg^2+^. In the presence of 333 μM ADP, 1 mM Ca^2+^ significantly (p˂0.001) increases O_2_ consumption, which decreases at a concentration of 10 mM Ca^2+^ (Fig. 8).

Repeating the measurements without the addition of mitochondria, we found that GSH did not result in O_2_ consumption.

## Discussion

**Fig. 9:**
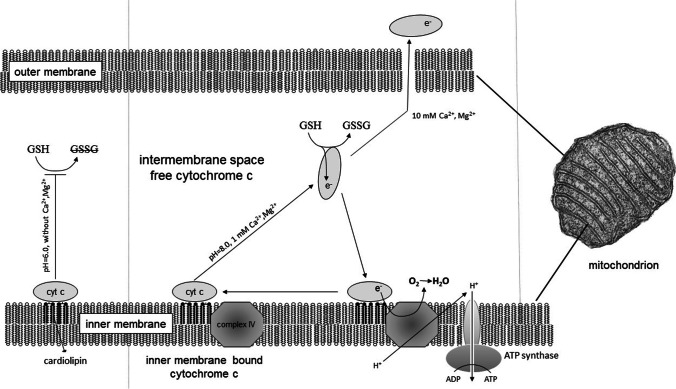
Summary. Putative mechanism of action. GSH: glutathione; GSSG: oxidized glutathione

In our work, we mainly characterized the reduction of cytochrome c by GSH by in vitro studies. In the experiments with mitochondria, O_2_ consumption was measured directly, and dissociation and reduction of cyt c from the membrane could not be monitored. Our hypothesis in Fig. 9 was based on our in vitro measurements as well as the cyt c pendulum mentioned above.

Our measurements also have methodological implications. The reduction of cyt c can be based on the measurement of superoxide dismutase activity (determination of superoxide concentration). Preeclamptic placenta can produce reactive oxygen species (Kukor and Valent, 2010). Depending on the physiological concentration of tetrahydrobiopterine (Sahin-Tóth et al. 1997 et al. 1997; Tóth et al. 1997), eNOS monomerizes-dimerizes and can produce NO or superoxide. According to our unpublished results (Kukor, Csomó, Valent), in the presence of DTT, Ca, Mg (1–1 mM) required for eNOS activity (Kukor and Tóth, 1994), the possible superoxide production of the placenta cannot be measured by following cyt c reduction due to the high background (reduction of cyt c by DTT).

In the presence of phospholipids, but in the absence of Ca^2+^ and Mg^2+^, GSH does not reduce cyt c. The cyt c reduction is most strongly inhibited by mitochondrial CL. There are several reasons for this effect. On one hand, they can promote dissociation of cyt c from the phospholipids. On the other hand, the divalent cations also increase the reduction of the free (without added phospholipid) cyt c. This suggests that Ca^2+^ and Mg^2+^ may directly affect the function of cyt c. Mitochondria uses GSH as a respiratory substrate at high pH, ​​in the presence of Ca^2+^ or Mg^2+^, while at pH 7.0, in the presence of EDTA, GSH does not increase the O_2_ consumption of mitochondria. Cyt c experiments suggest that GSH may be a substrate for mitochondrial respiration if cyt c dissociates from the inner membrane of the mitochondria.

Without ADP, Mg^2+^ does not increase mitochondrial O_2_ consumption. This means that O_2_ consumption can be linked to a respiratory chain (including cyt c). However, the Mg^2+^ requirement of O_2_ consumption, according to our measurements, may result in the cleavage of cyt c from phospholipids. Cyt c is present at high concentrations in the intermembrane space of intact mitochondria (up to 0.7 mM) at physiological intermembrane space ionic strength (Hackenbrock et al. 1986).

Our observation is explained by the fact that cyt c does not oxidize GSH at low Mg^2+^ concentrations. At higher (1 mM) concentrations of Mg^2+^, cyt c detaches from the membrane, allowing it to oxidize GSH. Cyt c remains in the intermembrane space, can be attached to the inner membrane, the electron can be transferred to O_2_. Several articles describe that cytochrome c can enter the intermembrane space from the inner membrane, be reduced, and then rebound to the inner membrane where it is oxidized by cyt c oxidase. This cycle also has a physiological role, participating in the formation of disulfide bonds in proteins (Endo et al. 2010; Cortese et al. 1998). At higher Mg^2+^ concentrations (10 mM), cyt c may be released from the mitochondria and therefore can no longer return to the inner membrane. This reduces mitochondrial O_2_ consumption (Fig. 9).

The binding of cyt c to CL causes a change in the redox properties of cyt c (Kagan et al. 2004). The tightly membrane-bound cyt c has one third of the electron transport capacity of the electrostatically bound cyt c. The CL bound cyt c would not be able to convert O_2_^.−^ to O_2_. The anionic lipids-bound cyt c blocks the electron transfer from a sulphydryl group to cyt c. Therefore, cyt c cannot oxidize glutathione. This affects energy saving, because the electron from NADH comes in to the electron transfer chain at complex I, while the electron of the –SH group comes in at complex IV. The oxidized glutathione that was reduced by glutathione reductase only works with NADH. Changes in the intracellular milieu of the cells, such as alterations in the redox environment, are important regulators of the progression to apoptosis (Pervaiz and Clement 2002). Depletion of GSH is an early hallmark in the progression of cell death in numerous cell types (Circu and Aw 2008, Circu and Aw 2012). The intrinsic mitochondrial apoptotic pathway can be activated by oxidative stress. Various apoptotic stimuli (e.g. reactive oxygen species) mediate permeabilization of the outer mitochondrial membrane and the release of proapoptotic proteins (cyt c, Apaf-1, endonuclease G). Based on our findings, we suppose that the release of cytochrome c in to the intermembrane space can rapidly decrease the GSH level in the mitochondrial intermembrane space. Cyt c concentration in the intermembrane space may be comparable to the level of GSH, 0.5–1.0 mM (Bayir et al. 2006). O_2_ consumption of the mitochondria increases at 1 mM GSH, pH 8.0, 1 mM Mg^2+^, thus reduced cyt c is reoxigenized by oxygen.

Physiological concentration of magnesium (1 mM) causes apoptosis in cesarian placentae tissue culture and apoptosis was reduced by antioxidant agents such as ascorbic acid and N-acetyl-cystein (Black et al. 2001). Serum magnesium concentrations are decreased in normal pregnancy and elevated in preeclampsia (Sanders et al. 1999). Thus it is worth considering the benefits and necessity of magnesium supplementation.

GSH depletion is a common feature of apoptotic cell death. Although previous studies suggested that GSH depletion was only a byproduct of oxidative stress generated during cell death, recent discoveries suggest that GSH depletion are critical regulators of apoptosis (Circu and Aw 2012). Some reports suggest that mitochondrial GSH depletion is important in triggering the cell death cascade (Circu and Aw 2012, Lash 2006, Brookes et al. 2004). It seems that the oxidation of GSH has an important role in the opening of the PTP in addition to the cyt c release (Constantini et al. 1996, Beatrice et al. 1984), and the Mg^2+^ dependent cyt c release (Eskes et al. 1998). Our recent work supposes that free cyt c oxidases GSH, thereby increasing oxidative stress in the mitochondria without reactive oxygen species.

## Abbreviations

Cyt c: Cytochrome c
DTT: Dithiothreitol
EDTA: Ethylenediamine tetraacetic acid
GSH: Glutathione
GSSG: Oxidized glutathione
PTP: Permeability transition pore
